# Evaluation of visual performance and eye movements in patients with blue light-filtering intraocular lenses versus ultraviolet light-filtering intraocular lenses

**DOI:** 10.3389/fnins.2023.1207853

**Published:** 2023-12-21

**Authors:** Yan Liu, Xiaotong Ren, Yu Wan, Luling Yang, Rong Zhang, Xuemin Li

**Affiliations:** ^1^Department of Ophthalmology, Peking University Third Hospital, Beijing, China; ^2^Beijing Key Laboratory of Restoration of Damaged Ocular Nerve, Peking University Third Hospital, Beijing, China; ^3^Department of Neurobiology, School of Basic Medical Sciences, Peking University, Beijing, China; ^4^Neuroscience Research Institute, Peking University, Beijing, China; ^5^Key Laboratory for Neuroscience, Ministry of Education/National Health and Family Planning Commission, Peking University, Beijing, China

**Keywords:** eye movement, blue light-filtering IOL, visual performance, everyday tasks, ultraviolet light-filtering IOL

## Abstract

**Background:**

Eye movement plays an important role in visual perception and provides essential visual information for everyday tasks. Our previous study indicated that the visual performance and eye movement pattern were impaired in age-related cataract patients and could be improved after cataract surgery, but the impact of different intraocular lens (IOL) types was obscure. Previous studies found that blue light might affect the eye movement pattern and cognitive function. In this study, we explored the visual performance and eye movement pattern in post-cataract surgery patients implanted with blue light-filtering IOLs or ultraviolet (UV) light-filtering IOLs to further understand the impact of different types of IOLs on and daily visual performance and eye movement pattern and to help ophthalmologists and patients make the personalized option of IOL types in future.

**Methods:**

Patients after both-eye cataract surgeries were included in this study. Eye movement behaviors were automatically recorded by an eye tracker while performing three performance-based everyday tasks (non-social object search, face recognition, and reading). Visual performance and eye movement parameters were compared between participants with blue light-filtering IOLs and UV light-filtering IOLs. The correlation between visual performance and eye movement parameters was explored to determine which eye movement parameters had a significant influence on visual performance outcomes.

**Results:**

A total of 30 patients (16 with blue light-filtering IOLs and 14 with UV light-filtering IOLs) were included. In this study, we found that the eye movement pattern was slightly different with these two IOLs: during non-social object visual search task, time to first fixation and fixation counts before first fixation were notably increased in yellow-tinted blue light-filtering IOL patients. During reading task, a higher total fixation count was also found in blue light-filtering IOL patients. However, the visual performance of these two IOLs was close, except for a quicker search of the target object with clear UV light-filtering IOLs.

**Conclusion:**

Both blue light-filtering and UV light-filtering IOLs were able to successfully restore visual function and yield satisfactory outcomes after cataract surgery. Although subtle, yellow-tinted IOLs did have a slight but significant impact on visual performance and the eye movement pattern of elderly patients when handling everyday tasks.

## Introduction

Eye movement is essential for human visual information input because of the limited high acuity of central vision. By frequently orienting our eyes to different points in the visual field, we could ensure satisfactory vision in areas of interest. Although peripheral vision is very useful in some real-world tasks such as driving and walking, the central vision could provide the most information with a high resolution (Gloriani and Schütz, 2019; Srikantharajah and Ellard, 2022). Eye movement, including fixations and saccades, which can be made voluntarily, is an essential component of visual function in performing everyday activities (Foulsham, 2015). Abnormalities in eye movement can be observed in a wide variety of neurological conditions, such as Parkinson’s disease, acute cerebral accident, degenerative vestibular disorders, and epilepsy (Xiaojun, 2008; Shaikh and Ghasia, 2019), which are comprehensively investigated in the neurophysiologic area.

Ocular disorders could also affect the eye movement pattern due to poor central vision or visual field defects (Stifter et al., 2004; Burton et al., 2014; Boucart et al., 2015; Thepass et al., 2015; Dive et al., 2016). Previous studies reported that patients with age-related macular degeneration (AMD) need longer gaze durations and more eye movements than normally sighted people when doing natural actions (such as making sandwiches or model building) (Boucart et al., 2015). Glaucoma patients were slower to perform daily tasks (the same activities above) with longer fixation times and more head and eye movements (Dive et al., 2016). Patients with advanced glaucomatous visual field (VF) defects read more slowly accompanied with changes of eye movement behavior (Burton et al., 2014). Many previous studies also found an alteration in visual performance and eye movements in patients with age-related cataract (Stifter et al., 2004; Thepass et al., 2015). Cataract surgery combined with intraocular lens (IOL) implantation proved to be a very effective way of restoring the visual function and improving visual performance in daily activities (Wan et al., 2020; Nowrouzi et al., 2023). Furthermore, our previous study found that the improvement of visual performance after cataract surgery was associated with the eye movement pattern changes post-operatively (Wan et al., 2020).

Blue light-filtering IOLs are commercially available and are widely used in cataract surgeries in recent decades (Mainster, 2006; Zhu et al., 2012; Downie et al., 2018). They were first introduced into ophthalmologic practice in 1991. Blue light-filtering IOLs are designed in yellow or orange to selectively attenuate the transmission of short-wavelength (about 400–500 nm) visible light for the aim of retina protection, especially for protecting macular health (Mainster, 2006). In contrast, UV light-filtering IOLs are colorless and mainly absorb UV radiation (wavelength 200–400 nm) and a small amount of violet light (wavelength 400–440 μm), while most blue light (wavelength 440–500 μm) could pass through freely (Mainster, 2006). The visual performance of patients implanted with blue light-filtering IOLs was assessed concerning visual acuity, contrast sensitivity, and color perception. Various previous studies found that postoperative visual performance (including visual acuity and contrast sensitivity) with blue light-filtering IOLs is approximately equal to that of UV light-filtering IOLs, but only some mild compromise in the blue light spectrum under mesopic light conditions was detected (Zhu et al., 2012; Downie et al., 2018). However, the impact of blue light-filtering IOLs on everyday task visual performance and eye movements remained obscure.

Previous studies found that blue light influences the eye movement pattern and enhances the speed of saccadic eye movements (Poth and Schneider, 2016; Lee and Yeh, 2021). Accordingly, we could reasonably infer that the blue light-blocking effect of yellow-tinted IOL implanted in the eyes might cause changes in the eye movement pattern and affect the performance of central vision-related daily activities (Tzamalis et al., 2020).

Most importantly, cataract surgery could have an impact on the eye movement pattern in elderly people, and it was related to the improvement of visual performance in daily activities (Tzamalis et al., 2020; Wan et al., 2020; Nowrouzi et al., 2023). Since different types of IOLs were used in clinical settings, including blue light-filtering IOLs, this study aimed to explore the eye movements and visual performance of cataract surgery patients implanted with blue light-filtering IOLs and UV light-filtering IOLs to further understand the eye movement pattern influenced by different types of IOLs during performance-based everyday tasks, including non-social object search, face recognition, and reading.

## Methods

### Study design and participants

An observational study was conducted between June 2019 and December 2019 in the Department of Ophthalmology, Peking University Third Hospital, Beijing, China. Patients who underwent both-eye, uncomplicated phacoemulsification combined with IOL implantation surgeries were included in this study. Patients were implanted with mono-focal-designed IOLs, either blue light-filtering ROHTO RAY-61PL IOL (Rohto Pharmaceutical Co., Ltd., Osaka, Japan) or UV light-filtering Tecnis ZCB00 IOL (Abbott Medical Optics Inc., Santa Ana, CA, United States). A comprehensive ocular examination was carried out, and any ocular comorbidity other than cataract (such as glaucoma, corneal abnormalities, or retinal diseases) was excluded from this study. Medical records were reviewed, and subjects with any recorded or self-reported systemic diseases, especially psychiatric or recognition disorders that might cause cognitive deficits (such as Alzheimer’s disease, Parkinson’s disease, acute cerebral accident, degenerative vestibular disorders, and epilepsy), were also excluded.

**Figure 1:**
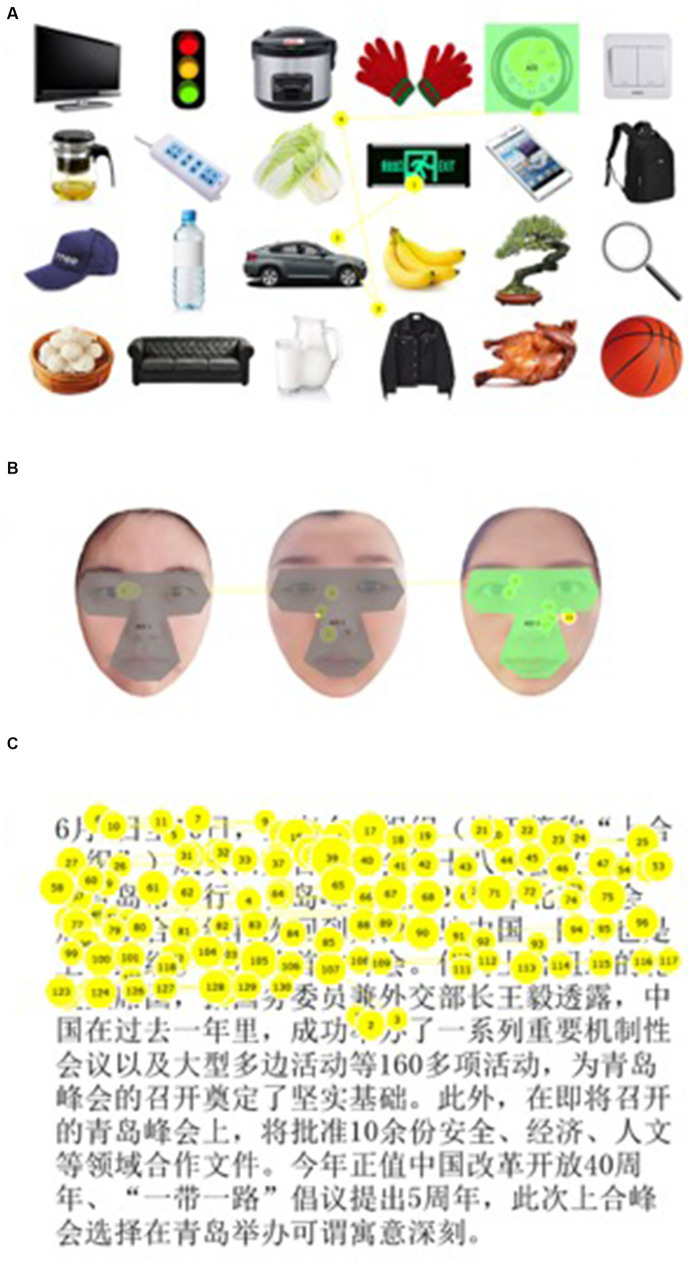
Examples of eye movement tracking tasks. **(A)** Example of non-social object search task. The gaze positions of fixations (yellow circles) and scan paths of saccades (yellow lines) made by a participant as he/she was asked to find a “clock” were marked out in the figure. The size of the circles corresponded to fixation duration. The number in the circles represented the rank of fixation. Area of interest (AOI) was marked in green color. **(B)** Example of face recognition task. This is an example of using the photos of researchers for illustration purposes only. Fixations and saccades were mapped in yellow color. The target AOI was illustrated in green color, which contained the key facial features, including the eyes, nose, and mouth. Gray color showed the invalid AOI. **(C)** Example of the reading task. This is an example of using the first passage in font size 28. The participant read sentences in a horizontal direction from left to right. The fixations were shown in circles with numbers, and the saccades were represented by lines.

The tests were conducted 3 months after cataract surgeries. Basic ocular examination included distant, medium, and near-best corrected visual acuity (BCVA), dynamic visual acuity with speeds of 1, 4, 8, and 12 m/s, and slit-lamp examination. The visual performance and eye movement pattern were evaluated during non-social object search, face recognition, and reading tasks by masked examiners. The study conformed to the tenets of the Declaration of Helsinki, and the procedures were reviewed and approved by the Peking University Third Hospital Medical Science Research Ethics Committee (approval number: 2018/282–02). Authors could have access to information that could identify individual participants during data collection, and written informed consent was obtained from all participants. In particular, informed consent for all images used in this study (the face images in Figure 1 were photos of researchers) was obtained for publication.

### Testing procedure and visual tasks

The eye tracking tests were conducted in a specific ophthalmic examination room with a constant ambient luminance. Participants were required to sit 65 cm from a 21.5-in computer monitor displaying at a resolution of 1920 × 1,200 (Dell ST2220Mb; Dell Corporation, Texas, United States). The eye tracker (Tobii Pro X3-120 Eye Tracker; Tobii AB Inc., Danderyd, Sweden) was fixed on the monitor to record eye movement data during the trials. As Tobii possessed excellent accuracy and precision with a high tolerance for head movements, we did not restrict the participants’ heads with stabilizer in order to reproduce the real visual conditions in daily life and thus capture more natural eye movement behaviors. Prior to the formal testing, each participant was given a full and detailed explanation of every procedure of the test. All individuals were tested under binocular viewing conditions with best spectacle-corrected vision.

The whole test consisted of three everyday task parts: (1) non-social object visual search; (2) face recognition; and (3) reading task. Detailed testing procedure was illustrated in the previous study (Wan et al., 2020). The pictures or texts used in object search (Figure 1A), face recognition (Figure 1B), and reading tasks (Figure 1C) are shown in Figure 1.

The whole eye tracking tests were completed twice for each participant. To minimize the influence of learning effects, the testing procedure remained the same, but all stimuli were replaced by new items, faces, and passages in the second assessment. When doing the non-social object search tests, different sizes of objects were asked to be searched to decrease the influence of varying sizes of the objects. Average values of the two tests were recorded as result data.

### Eye movement parameters

Eye movements were tracked with the Tobii Pro X3-120 Eye Tracker with an accuracy of 0.4° and a sampling rate of 120 Hz. Once the participant was correctly set up, an automated calibration was started by ToolBox software. The eye tracking data were automatically recorded in the Tobii Studio eye tracking software (Tobii AB, www.tobii.com) during testing. Fixations were defined by the default fixation algorithm for Tobii Studio as stable gazes with positions remaining for at least 100 ms. Saccades were defined as the eye movements that occur between fixations (Lee et al., 2015). To evaluate the gaze behaviors, AOIs were marked out in each image using the data tools available in the Tobii Studio program. AOIs were defined using a square shape around the target object or an irregular shape surrounding the key facial features (eyes, nose, and mouth) of the target face (Figure 1). The participants were asked to keep fixating on the target object after he/she found it in the visual search and face recognition tasks; each successful identification was detected by analyzing the time series of his/her eye movement. If a fixation was located on the AOI of the target, followed by a series of gazes within the AOI, it was regarded as a successful identification. A failed identification did not have successive fixations remaining within the AOI.

Eye movement parameters included time to first fixation, fixation counts before first fixation, first fixation duration, mean fixation duration, total fixation counts, total fixation duration, and total visit duration. The definition of each parameter is described in Table 1. If, at the end of the recording, the participant had not fixated on the AOI, the above metrics would not be computed, and thus, that recording would not be included in the statistics calculations.

**Table 1:** Definition of eye movement parameters.

Eye movement parameters	Definition
Time to first fixation(s)	How long it take before the participant fixates on an AOI for the first time?
Fixation counts before first fixation	How many fixations occur before the participant fixates on an AOI for the first time?
First fixation duration(s)	The duration of the first fixation within an AOI
Mean fixation duration(s)	The mean duration of the fixations within an AOI
Total fixation counts	Total number of times the participant fixates on an AOI
Total fixation duration(s)	The sum of the duration for all fixations within an AOI total fixation duration = mean fixation duration × total fixation counts
Total visit duration(s) or observation length(s)	The sum of the duration for all visits within an AOI, including fixations as well as saccades

### Visual performance

Visual performance of participants was evaluated by correctly identified percentage (%) and average search time(s) for non-social objects and face identification tests. The percentage of correctly identified objects/faces was calculated by dividing the number of successful identifications by the number of trials. Search time was considered equal to “time to first fixation.” Reading speed was used to assess visual performance in reading tests, which was calculated as the total number of characters divided by total visit duration (chars/s). Visual performance of participants with these two types of IOLs was compared and analyzed. Further analysis was conducted to determine the correlation between visual performance and eye movement parameters.

### Statistical analysis

Statistical analyzes were performed using SPSS version 23 (IBM Corp., Armonk, NY, United States) and GraphPad Prism version 8 (GraphPad Software Inc., San Diego, CA, USA). Based on our previous study, our primary outcome parameter percentage of correctly identified items was 85.19 ± 9.90% post-cataract surgery. Using a two-sided level of significance (α) of 5% and power (1 − β) of 80%, a sample size of 15 patients was required to detect a difference of 10% in the percentage of correctly identified items between groups. Comparisons of eye movement parameters and visual performance between blue light-filtering IOLs and UV light-filtering IOLs were done using independent Student’s *t*-tests (when the variables were normally distributed) or by the Wilcoxon signed-rank test (when the variables were not normally distributed). Spearman’s correlation analyzes were used to evaluate associations between eye movement parameters and visual performance outcomes. The Spearman correlation coefficient of less than 0.2 was considered no relationship, whereas values of 0.2–0.4 suggested a weak relationship; those from 0.4 to 0.6 indicated a moderate relationship; and values of 0.6–0.8 indicated a strong relationship. Multiple linear regression analyzes with stepwise selection (a *p*-value of <0.05 as the selection criterion) were used to determine which eye movement parameters had significant influence on visual performance outcomes. The factors included in the multiple linear regression analyzes were age and each eye movement parameter as independent variables and visual performance as a dependent variable (total visit duration was excluded in the multiple linear regression analysis because of its notable collinearity with total fixation duration).

## Results

### Participants

This study included 30 subjects who underwent cataract surgeries 3 months ago (16 with blue light-filtering IOLs vs. 14 with UV light-filtering IOLs). There were 11 male and 19 female subjects who had a mean age of 70.11 ± 7.36 years (range 57–80 years). The demographic information and visual function characteristics of the participants are presented in Table 2. The distant, medium, and near-corrected visual acuity was not significantly different between the two groups. Dynamic visual function tests showed dynamic visual acuity under different movement speeds, which were also statistically identical in the two groups. One point that needs to be emphasized is that the subjects involved in this study were a new group of participants besides participants involved in the previous study.

**Table 2:** Demographic information and visual function of participants.

	Blue light-filtering IOL group (*n* = 16)	UV light-filtering IOL group (*n* = 14)	*p*
Age (years)	69.94 ± 7.89 (range 57–80)	70.40 ± 6.75 (range 57–77)	0.879
Sex (male/female)	6/10	5/9	0.919
Distant VA	0.13 ± 0.02	0.12 ± 0.02	0.365
Medium VA	0.46 ± 0.02	0.48 ± 0.03	0.443
Near VA	0.50 ± 0.03	0.49 ± 0.02	0.258
Dynamic VA (1 m/s)	0.249 ± 0.021	0.252 ± 0.019	0.645
Dynamic VA (4 m/s)	0.335 ± 0.018	0.376 ± 0.024	0.387
Dynamic VA (8 m/s)	0.356 ± 0.032	0.338 ± 0.026	0.526
Dynamic VA (12 m/s)	0.406 ± 0.025	0.423 ± 0.033	0.432

VA represents visual acuity. Visual acuity in this table was shown in logMAR format.

### Visual performance of the blue light-filtering IOL and UV light-filtering IOL groups

In non-social object search task, the percentage of correctly identified objects in the blue light-filtering IOL group and the UV light-filtering IOL group was 80.42 and 78.33% (*p* = 0.796), respectively, which indicated similar accuracy of non-social object recognition ability among subjects with yellow or clear IOLs. However, the average search time of clear IOLs was significantly shorter than that of yellow IOLs (1.00 ± 0.49 s vs. 1.45 ± 0.51 s, *p* = 0.027), indicating that subjects with UV light-filtering IOLs could identify the target object faster than those with blue light-filtering IOLs.

In face recognition task, the correctly identifying percentage (82.50% vs. 88.33%, *p* = 0.482) and average search time (0.99 ± 0.57 s vs. 1.10 ± 0.83 s, *p* = 0.688) were not significantly different in these two groups, indicating a similar face recognition ability between subjects with yellow or clear IOLs.

In reading task, reading speed was 5.94 ± 2.62 chars/s and 6.42 ± 2.52 chars/s in the blue light-filtering and UV light-filtering groups, respectively. Participants read slightly faster with clear IOLs, but the difference was not significant (*p* = 0.175). Visual performance of the three everyday tasks is shown in Table 3.

**Table 3:** Visual performance of patients with blue light-filtering IOLs and UV light-filtering IOLs.

	Blue light-filtering IOLs		UV light-filtering IOLs		*p*
	Mean ± SD	Range	Mean ± SD	Range	
*Non-social object search*					
Correctly identified percentage (%)	80.42 ± 18.59	41.67–100	78.33 ± 24.28	25.00–100	0.796
Average search time(s)	1.45 ± 0.51	0.53–2.47	1.00 ± 0.49	0.37–1.79	0.027*
*Face recognition*					
Correctly identified percentage (%)	82.50 ± 22.60	33.33–100	88.33 ± 17.66	50.00–100	0.482
Average search time(s)	0.99 ± 0.57	0.17–2.05	1.10 ± 0.83	0.11–2.36	0.688
*Reading task*					
Reading speed (chars/s)	5.94 ± 2.62	2.56–9.91	6.42 ± 2.52	1.92–9.60	0.634

IOL represents intraocular lens.

* means that significant difference was detected between blue light-filtering and UV light-filtering IOLs.

### Eye movement parameters of the blue light-filtering IOL and UV light-filtering IOL groups

Accordingly, in non-social object search task, time to first fixation was shorter, and fixation counts before first fixation were lower in the UV light-filtering group. Other eye movement parameters (including fixation counts, first fixation duration, mean fixation duration, total fixation duration, and total visit duration) were not significantly different between subjects with yellow or clear IOLs. In face recognition task, consistent with visual performance, there were no notable differences in all eye movement parameters between the two types of IOLs. In reading task, we found fixation counts with UV light-filtering IOLs were less than those with blue light-filtering IOLs (60.30 ± 17.16 vs. 74.26 ± 15.15, *p* = 0.035), which might be related to reading performance. The differences between other eye movement parameters were not significant (*p* > 0.05). All the eye movement parameters are shown in Table 4.

**Table 4:** Eye movement parameters of patients with blue light-filtering IOLs and UV light-filtering IOLs.

	Blue light-filtering IOLs	UV light-filtering IOLs	*p*
*Non-social object search*			
Time to first fixation(s)	1.45 ± 0.51	1.00 ± 0.49	0.027*
Fixation counts before first fixation	4.58 ± 1.59	2.52 ± 1.46	0.002*
First fixation duration(s)	0.31 ± 0.16	0.36 ± 0.31	0.607
Mean fixation duration(s)	0.43 ± 0.22	0.44 ± 0.38	0.867
Total fixation counts	5.06 ± 1.93	5.54 ± 2.09	0.534
Total fixation duration(s)	1.85 ± 0.68	2.06 ± 1.14	0.542
Total visit duration(s)	2.17 ± 0.82	2.44 ± 1.36	0.572
*Face recognition*			
Time to first fixation(s)	0.99 ± 0.57	1.10 ± 0.83	0.688
Fixation counts before first fixation	2.84 ± 1.69	2.50 ± 1.88	0.612
First fixation duration(s)	0.21 ± 0.07	0.27 ± 0.15	0.321
Mean fixation duration(s)	0.29 ± 0.13	0.35 ± 0.19	0.316
Total fixation counts	5.20 ± 2.44	5.16 ± 2.41	0.961
Total fixation duration(s)	1.49 ± 0.93	1.89 ± 1.25	0.333
Total visit duration(s)	1.78 ± 1.13	2.20 ± 1.52	0.393
*Reading task*			
Time to first fixation(s)	1.15 ± 1.63	0.90 ± 0.95	0.663
Fixation counts before first fixation	2.58 ± 5.12	1.13 ± 1.29	0.389
First fixation duration(s)	0.18 ± 0.04	0.22 ± 0.08	0.072
Mean fixation duration(s)	0.24 ± 0.06	0.24 ± 0.05	0.976
Total fixation counts	74.26 ± 15.15	60.30 ± 17.16	0.035*
Total fixation duration(s)	16.76 ± 5.19	14.72 ± 6.71	0.371
Total visit duration(s)	24.01 ± 5.70	21.81 ± 8.30	0.407

IOL represents intraocular lens.

* means that significant difference was detected between blue light-filtering and UV light-filtering IOLs.

### Correlation of visual performance and eye movement parameters

The Spearman correlation analysis was used to determine the correlation between visual performance and eye movement parameters when handling these three everyday tasks. In non-social object search task, the correctly identified percentage was moderately correlated with mean fixation duration with a correlation coefficient (CC) of 0.47 (*p* = 0.008) and a total fixation duration with a CC of 0.40 (*p* = 0.029). Non-social object search time was positively correlated with fixation counts before first fixation (CC = 0.90, *p* < 0.001), negatively correlated with total fixation duration (CC = −0.51, *p* = 0.004), fixation counts (CC = −0.55, *p* = 0.002), and total visit duration (CC = −0.58, *p* = 0.001). In face recognition test, the percentage of correct recognition was significantly correlated with mean fixation duration (CC = 0.39, *p* = 0.034), total fixation duration (CC = 0.69, *p* < 0.001), fixation counts (CC = 0.61, p < 0.001) and total visit duration (CC = 0.68, *p* < 0.001). Similarly, face search time was positively correlated with fixation counts before first fixation (CC = 0.89, *p* < 0.001), negatively correlated with mean fixation duration (CC = −0.44, *p* = 0.015), total fixation duration (CC = −0.65, *p* < 0.001), fixation counts (CC = −0.69, *p* < 0.001), and total visit duration (CC = −0.68, *p* < 0.001). In the reading test, reading speed was moderately correlated with total fixation counts (CC = 0.41, *p* = 0.031).

**Figure 2:**
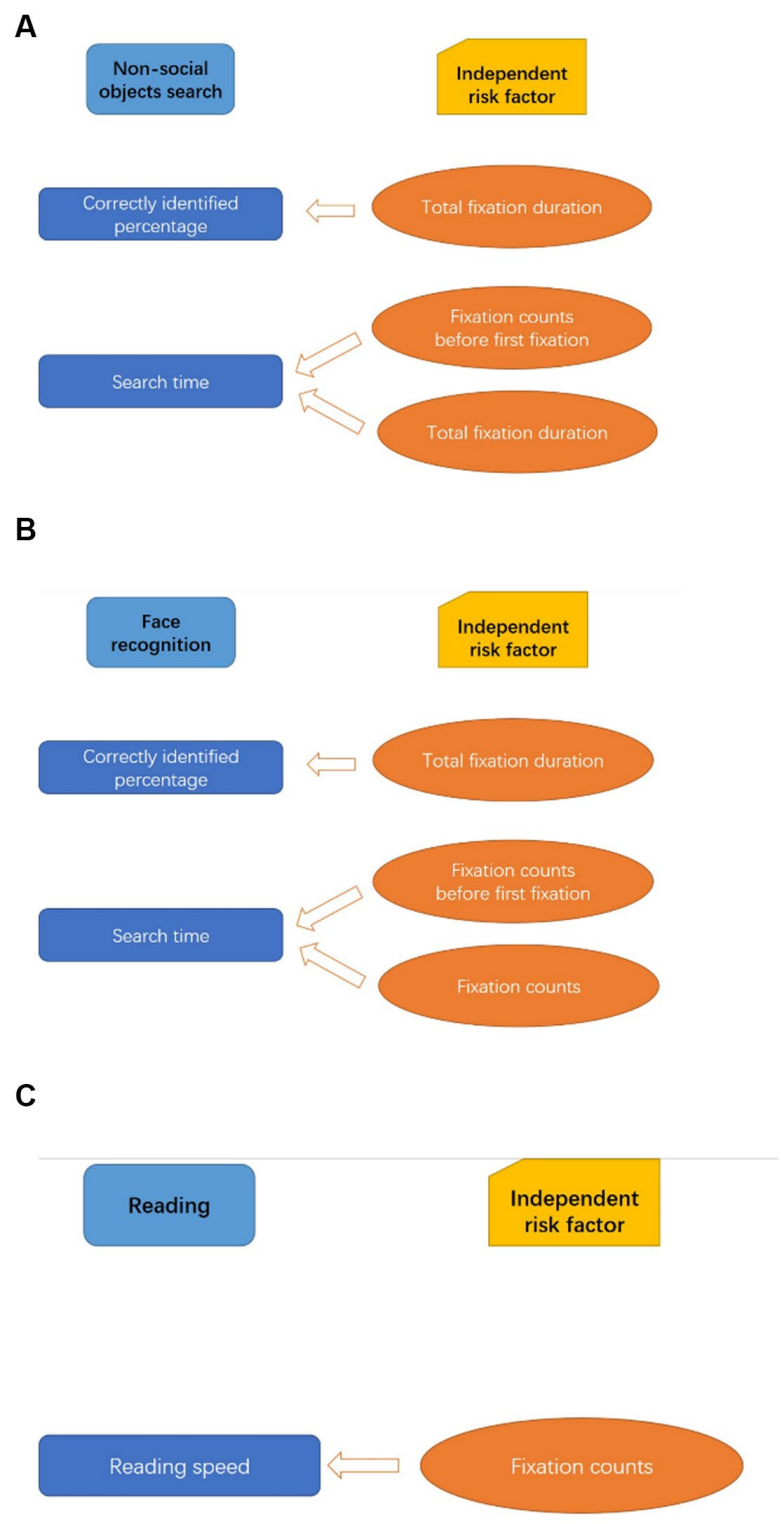
Independent risk factors for visual performance during three everyday tasks. **(A–C)**, respectively, demonstrate the results of multiple linear regression analyzes in a direct way. **(A–C)** show the independent risk factors for visual performance in each visual task.

Further multiple linear regression analyzes included age and eye movement parameters as independent variables and visual performance as a dependent variable. Analyzes showed that total fixation duration was an independent risk factor for non-social object identification (B = 1.22, *p* = 0.017). Fixation counts before first fixation (B = 0.22, p < 0.001) and total fixation duration (B = −0.16, *p* = 0.011) were independent risk factors for average search time. Similarly, total fixation duration was found to be an independent risk factor for face recognition accuracy (B = 0.75, p = 0.001), and fixation counts before first fixation (B = 0.27, *p* < 0.001) and fixation counts (B = −0.07, *p* = 0.035) were independent risk factors for face search time. Finally, in the reading task, an independent risk factor for reading speed was total fixation counts (B = 0.07, *p* = 0.020). Figure 2 shows the correlation between visual performance and eye movement parameters according to these results.

## Discussion

Our previous study investigated how the cataract surgery would influence the eye movement pattern and visual performance, and we found that elderly people with age-related cataract experienced an overall improvement in visual performance postoperatively, which is associated with the eye movement parameters altering after cataract surgery (Wan et al., 2020). This study was performed based on our previous study, and the purpose of this study mainly focused on the comparison of the eye movements and visual performance of two different types of IOL. This current study revealed that the eye movement pattern and daily visual performance of different types (mainly colors) of IOLs implanted in cataract surgery were similar, with a slight but significant difference in average search time before locating the non-social target (faster with clear IOLs), and this difference might be related to eye movement pattern changes. Although subtle, we found that blue light-filtering IOLs (yellow IOLs) might influence the non-social object identification process (with slower identification but similar accuracy) and might be related to reading habit alternation (increased fixation counts), but blue light-filtering IOLs basically have no impact on face recognition in daily life. The study design was exploratory research, and the objective of the study was to further understand the eye movement pattern influenced by different types of IOLs during performance-based everyday tasks and to help ophthalmologists and patients to make the personalized option of IOL types in future.

Many previous studies have explored the effects of different types of IOLs on visual performance and vision-related quality of life (Javitt and Steinert, 2000; Espindle et al., 2005; Xu et al., 2017). Novel IOLs, such as multifocal IOLs, aspheric IOLs, and blue light-filtering IOLs, were widely and thoroughly investigated (Javitt and Steinert, 2000; Espindle et al., 2005; Xu et al., 2017). It was reported that blue light-filtering IOLs might have potential benefits in glare reduction and protection against retinal phototoxicity (Davison et al., 2011) and could improve color vision, driving, and other aspects of health-related functioning and quality of life (Espindle et al., 2005). However, few studies focused on the effect of blue light-filtering IOL on the eye movement pattern. Our previous study found significant eye movement pattern changes and improved visual performance after cataract surgeries (Wan et al., 2020). Considering that blue light would influence the eye movement pattern and might enhance the speed of saccadic eye movements (Poth and Schneider, 2016; Lee and Yeh, 2021), the blue light blocking effect of yellow-tinted IOL might cause slowed saccadic eye movements, which was consistent with the results of this study with more average searching time before locating the non-social target. To the best of our knowledge, the present study is the first to investigate the effect of IOL type on eye movement pattern and further explore its possible relationship with visual performance in daily activities. This study found that, although visual acuity of different distances and dynamic acuity of various speeds were identical, the eye movement patterns slightly differed with blue light-filtering and UV light-filtering IOLs. In addition, it might, to some extent, have an impact on visual performance when searching for objects or reading in daily life.

Various studies confirmed that visual performance in daily activities was closely related to eye movement behaviors (Fooken et al., 2016; Paeye et al., 2016; Annac et al., 2019; Ramey et al., 2019). Some ocular diseases will impair vision function and affect eye movement pattern, but the results are often inconsistent and contradictory (Coeckelbergh et al., 2002; Kanonidou et al., 2010; van der Stigchel et al., 2013; Kanonidou et al., 2014; Taylor et al., 2017; Lee et al., 2019). Shorter fixation durations were reported in glaucoma patients compared to healthy controls (Coeckelbergh et al., 2002; Lee et al., 2019), while in some other studies, extended fixation durations were detected in strabismic amblyopic patients (Kanonidou et al., 2010, 2014). Moreover, some investigations of eye movements in AMD patients reported fixation durations on targets were longer (van der Stigchel et al., 2013); however, others found no change (Taylor et al., 2017). In this study, we found that fixation durations were not notably different with blue light-filtering and UV light-filtering IOLs, and fixation counts slightly increased with blue light-filtering IOL only in the reading task. This result indicated that the effect of IOL color might be subtle in fixation ability. In contrast, time or fixation counts before first fixation were significantly extended with blue light-filtering IOL, indicating a longer searching course before object identification. Although reading speed was statistically identical with these two IOLs, we found that it was moderately correlated with fixation counts, which was the only significantly different eye movement parameter between yellow and clear IOLs. This means that the eye movement mode does have an impact on reading and visual performance, consistent with many previous studies (Calabrèse et al., 2014; Kanonidou et al., 2014; Zeri et al., 2018). As stated above, eye movement behavior during daily activities is a complex process integrated with neuromotor, cognitive, visual, and oculomotor function coordinations. It was complicated to interpret the relationship between the eye movement pattern and visual performance.

In cataract patients, the lens opaqueness impairs the visual function in multiple ways, simultaneously altering the eye movement pattern (Thepass et al., 2015; Wan et al., 2020). Cataract surgery combined with intraocular lens implantation removes the opaque lens and replaces it with an artificial IOL. After cataract surgery, the patients have to readapt the “new” IOL. This process involves neurophysiological adaptation and visual function rebuilding and often yields a satisfactory outcome. Different types of IOLs might affect the post-surgery eye movement pattern reformation. The present study found changes in eye movement pattern during non-social object searching and reading but no changes in face recognition. The reasons might be multiple: First, the test design of face recognition task was less difficult—the participant was asked to identify the familiar face out of three options, while he or she was asked to identify the target object out of 24 items in the object search test. Second, compared to object items, face images appeared bigger, which would be helpful for participants to focus on. Above all, large numbers of studies demonstrated that people used different cerebral functional zones to process face recognition and object perception (Kanwisher et al., 1997; Caldara et al., 2003). Although it remains unclear how the divergence in brain cortical areas responsible for processing these stimuli directly elucidates the disparities observed, participants might exhibit heightened sensitivity to faces rather than non-social objects. In summary, detecting eye movement pattern discrepancies between yellow-tinted IOLs and clear IOLs help us gain more insights into the oculomotor behaviors during daily activities and thus better understand the effect of IOL types on the ability to handle daily business in the elderly population.

## Limitations

Inevitably, there are some limitations to this present study. First, although the sample size was determined by reference to previous similar studies, the sample size of participants was relatively small, which might not thoroughly reveal the differences between the two groups. One of the reasons is that the test procedure is relatively complex and requires superior cognitive function. Some elderly participants might not have completed the whole test and were excluded from this study. This situation also leads to a potential bias in our inclusion population. Most of them are well educated and come from an intellectual community. Consequently, the findings of this study might not be applicable to all individuals. In addition, the influence of sex on visual perception and eye movement patterns deserves attention (Shaqiri et al., 2018) and it will be further explored in larger cohorts, including different populations, in our future research. Second, the eye movement tracking device can only provide two-dimensional eye movement information, and the parameters provided by the machine are limited. Eye movement parameters adopted in this study mainly focus on the fixation function rather than saccadic eye movement, which may not sufficiently reflect the eye movement pattern in real life. Third, in the reading task, reading speed varies a lot between different individuals, which is often influenced by multiple factors, such as education level and personal experience (Calabrèse et al., 2014). Reading speed of participants in this study might be partly attributed to eye movement mode, but it can also be affected by other factors. We admit that eye movement is not the only influential factor in reading speed. Finally, this study only investigated yellow-tinted and clear IOLs, which was not sufficient to understand the effect of different types of IOL on eye movement pattern and real-life vision performance. Other types of IOL (such as multifocal, extended depth-of-field, or aspheric IOLs) were left for further investigation.

## Conclusion

Blue light-filtering IOL has a mild but detectable effect on eye movement pattern and everyday task visual performance. Performance-based visual function was correlated with eye movement behaviors in subjects with pseudophakic eyes. Our findings provide a new insight into the impact of IOL type on eye movement behavior in central vision-related daily activities and help us better understand the visual performance of different types of IOL.

## Data availability statement

The raw data supporting the conclusions of this article will be made available by the authors, without undue reservation.

## Ethics statement

The studies involving humans were approved by Peking University Third Hospital Medical Science Research Ethics Committee. The studies were conducted in accordance with the local legislation and institutional requirements. The participants provided their written informed consent to participate in this study.

## Author contributions

YL and XR conducted the experiments, analyzed the data, wrote the article, and edited the manuscript. YW and LY conducted the experiments and reviewed and edited the manuscript. XL and RZ conceived and designed the study, conducted the experiments, and reviewed the manuscript. All authors contributed to the article and approved the submitted version.
